# Highly affordable vaccines are critical for our continued efforts to reduce global childhood mortality

**DOI:** 10.1080/21645515.2019.1605817

**Published:** 2019-05-17

**Authors:** Stefan H.E. Kaufmann

**Affiliations:** a Department of Immunology, Max Planck Institute for Infection Biology, Berlin, Germany; b Hagler Institute for Advanced Study, College of Veterinary Medicine and College of Medicine, Texas A&M University, College Station, USA

**Keywords:** Vaccine preventable diseases, vaccines, supply, morbidity, mortality, prevention, serum institute of India, affordable price

## Abstract

Infectious diseases remain a major health threat, not only in resource-poor countries but also in pockets of poverty within middle-income and sometimes high-income countries. Whilst strong research and development for novel vaccines are urgently needed, equal care needs to be taken that current vaccines are produced at affordable prices so that universal childhood immunization will be accomplished. The Serum Institute of India (SII) has become the largest producer of affordable vaccines. Provision of SII produced vaccines against measles, rubella and meningitis to 73 GAVI supported countries alone will avert more than 5 million deaths between 2001 and 2020. Similarly, the SII produced measles vaccine, supplied to UNICEF and PAHO, can be attributed to nearly 22 million averted deaths between 1990 and 2016. Data presented provide compelling evidence for the crucial impact of partnerships between affordable vaccine producers and governmental, intergovernmental and nongovernmental organizations on universal vaccination to reduce childhood mortality.

## Introduction

Infectious diseases remain a major health threat which in numerous resource-poor countries are leading causes of mortality and morbidity. Overall, infectious diseases are on the decline, majorly because of vaccination^1^ but the situation has been severely aggravated by the increasing emergence of antimicrobial resistance (AMR) which is considered to be responsible for an estimated 700,000 deaths globally.^2^ AMR is not only on the rise in resource-poor but also in resource-rich countries.^2^ This has led to increased awareness about AMR and mobilization of numerous initiatives to develop novel antimicrobial drugs. Whilst new drugs are urgently needed, they will not resolve the issue of antibiotic resistance. Hence, on the long run, satisfactory control of infectious diseases requires additional measures including improved hygiene, rapid diagnosis and most of all a broader arsenal of vaccines. Hence, research and development (R&D) is critical for better intervention methods, to counter the continued threat of infectious diseases.^3-6^

Vaccines are amongst the most impactful medical achievements of the 20^th^ century and amongst the most cost-efficient medical intervention measures.^7,8^ Strong R&D efforts are needed for vaccines against diseases which still cannot be controlled by immunization. At the same time, we need affordable vaccines against those diseases which can already be controlled. To promote the core message that immunization of every child is vital to prevent diseases and protect life, the World Immunization Week will take place April 23–29, 2019.^9^ Here I describe how the close cooperation of the largest producer of vaccines, Serum Institute of India (SII), with governmental, intergovernmental and nongovernmental organizations has upheld global immunization programs at affordable cost. This is promising news on the background of many vaccines from big pharma companies becoming unaffordable in middle income and low income countries^10^

## SII contribution in the field of immunization

Since the first vaccine ever was conceived by Edward Jenner in 1796 against smallpox,^11^ vaccine development progressed by leaps and bounds. Most accomplishments occurred in the North; in the South, only few public sector vaccine manufacturing units existed, which produced a limited portfolio of vaccines including rabies, cholera, and diphtheria-tetanus-pertussis (DTP).^12^ Before the 1970s, tetanus was a major public health issue in India. Because of limited availability of tetanus toxoids (TT) vaccines, coverage was low and India was yet to start its expanded program of immunization (EPI). As a result, anti-tetanus serum (ATS) for passive vaccination was required in large quantities. However, it had to be imported and therefore it was in short supply and exceedingly expensive.

In 1966, Cyrus S. Poonawalla, a young commerce graduate decided to address this issue. In the same year, he founded SII and since then has navigated the institute successfully.^13^ SII started with ATS generated in horses from his stud farm. The product received profound demand and SII became a major supplier of ATS. In 1971 SII developed its first TT vaccine for active immunization. Due to this accomplishment, shortage of TT vaccines in India could be overcome. As a next step, DTP vaccine production began in 1973.

## SII Vaccines

Over the last 50 years, SII developed numerous vaccines including TT, diphtheria/tetanus toxoid (DT), tetanus reduced dose diphtheria toxoid (Td), diphtheria/tetanus/pertussis (DTP), measles, rubella, measles/rubella (MR), measles/mumps/rubella (MMR), bacille Calmette-Guérin (BCG), rabies, hepatitis B (HepB), *Haemophilus influenzae B* (Hib), DTP-HepB, DTP-Hib, DTP-HepB-Hib, meningococcal A conjugate (MenAfriVac), pandemic influenza H1N1, trivalent live attenuated seasonal influenza, rotavirus, inactivated polio (IPV), trivalent oral polio (tOPV), and bivalent oral polio (bOPV). Except for the last three vaccines, these products were manufactured within SII from bulk stage. tOPV and bOPV manufacturing was based on the bulk received from PT Biofarma, Indonesia and IPV from Bilthoven Biologicals, the Netherlands .

## SII vaccine supplies to various agencies

In 1978, the Government of India started the EPI which in 1985 was renamed Universal Immunization Programme (UIP). It included BCG, OPV, DPT, TT and measles vaccines. HepB vaccine, the second dose measles vaccine and the pentavalent DTP-HepB-Hib vaccine became part of the UIP in last 20 years. Measles vaccine catch-up campaigns were conducted in few states of India during 2010 to 2012,^12^ and in 2017, the MR immunization campaign was launched in India. In the same year, the MR vaccine replaced the measles vaccine at both age groups, 9 months and 15 months, and became part of UIP.^14^ In the last 20 years the states of Delhi, Goa, Sikkim, and Pondicherry introduced the MMR vaccine in their UIP schedules.^15^ With an annual birth cohort of 27 million, the Indian immunization programme is the largest in the world.^16^

Since the launch of EPI in 1978 SII has been a major supplier for numerous vaccines including DPT, DT, TT, Td, DTP-HB-Hib, measles, MR, MMR and BCG to governmental immunization programmes. In addition, SII supplied a significant proportion of rabies vaccines to Central and State Hospitals for patients of animal bites.

United Nations agencies including United Nations International Children‘s Emergency Fund (UNICEF) and Pan American Health Organization (PAHO) procure vaccines and UNICEF supplies vaccines to developing countries in Asia, Africa, and Eastern Europe while the focus of PAHO is on most of the countries in Latin America. Only vaccines that are prequalified by the World Health Organization (WHO) are eligible for procurement by UNICEF and PAHO.^17^ Prequalification by WHO is made after a rigorous scrutiny of regulatory dossier, manufacturing site inspection and testing of vaccine samples by independent laboratories.

As the first prequalified vaccine by any developing country vaccine manufacturer, SII’s measles vaccine was prequalified by the World Health Organization (WHO) in 1992, followed by SII’s DTP vaccine in the subsequent year. Since then, a total of 26 SII vaccines (maximum by any manufacturer) have been prequalified by WHO including TT, DT, Td, DTP, HepB, Hib, DTP-HepB, DTP-Hib, DTP-HepB-Hib, measles, rubella, MR, MMR, BCG, pandemic H1N1, trivalent seasonal influenza, MenAfriVac, IPV, bOPV, tOPV. Since 1992 SII has been supplying large quantities of vaccines to UNICEF and PAHO for distribution in the respective regions.

India’s health-care system involves not only governmental health-care institutes but also a large private health-care sector. The latter comprises private hospitals as well as clinics of practicing physicians. It has been estimated that around 2.3% to 7.6% of the children receive their EPI vaccines from the private sector.^18^ Since its beginning, SII has supplied large quantities of its vaccines to the private health-care sector. Also in other countries, the private health-care sector participates to varying degrees and after the first prequalification in 1992, SII started exporting vaccines to other countries. Since then numerous SII vaccines have been registered in various countries and SII has supplied huge quantities of vaccines to foreign private markets in Asia, Africa, Latin America and countries of the Commonwealth of independent states (CIS) of the former Soviet Republic. In addition, several governments which are not eligible for supplies from UNICEF or PAHO procure vaccines through national tenders. Even countries which are eligible for UNICEF or PAHO supplies sometimes procure vaccines according to their local needs from SII. Vaccines from SII are mainly used for routine immunization programs in these countries. In several instances, the vaccines were also provided for mass immunization campaigns (either local, regional or national). For example, many countries in Latin America introduced the MMR vaccine in routine immunization and conducted mass immunization campaigns with the MR vaccine. These efforts culminated in the elimination of measles in 2016 and the elimination of rubella and congenital rubella syndrome in 2017 in the whole of Latin America.^19^

In a similar vein, since 2010 the MenAfriVac campaigns were conducted in several Sub-Saharan countries known as Meningitis belt culminating in the elimination of meningitis A from these countries.^20^ Nationwide campaigns with the MR vaccine conducted in several countries significantly impacted on the morbidity and mortality due to these two infections. In most cases, the MR vaccine was supplied by SII because it is the only prequalified MR vaccine globally.^21-24^

## Statistics on doses supplied

In the following, numbers of vaccine doses supplied by SII and their impact on global mortality due to the respective diseases were estimated. Although morbidities must be several folds higher, it was not feasible to assess vaccine impact on morbidity because reliable global data on morbidity due to these diseases are not available and thus focus was given to averted deaths.

Table 1 depicts the year of licensing and the year of prequalification of different SII vaccines. Since vaccine antigens are present in various combinations, the first licensing and prequalification are given (e.g., Diphtheria is present in DT, DTP, DTP-HB, DTP-Hib, and DTP-HB-Hib, but only the year in which the antigen was licensed for the first time was considered). Table 1 also provides numbers of doses distributed globally until 31 March 2018 and estimated number of individuals fully vaccinated.

**Table 1.** Total numbers of SII vaccine doses distributed globally and estimated numbers of individuals fully immunized for each vaccine antigen.^a, b^

Antigen	Year licensed	Year prequalified	Number of distributed doses (in millions) until 31 March 2018	Number of individuals fully immunized (in millions)
Rabies (HDC + Vero cell based)	2002		9.60	1.92
Diphtheria (D + reduced dose d)	1974	1995	4288.66	1429.55
Pertussis	1974	1995	3258.86	1086.29
Tetanus	1972	1995	7650.80	2550.27
Hepatitis B (20 IU + 10 IU)	2000	2004	1245.89	415.29
Hib	2007	2008	821.40	273.8
Polio (tOPV + bOPV + IPV)	2013	2013	1848.72	616.24
Rotavirus	2017	2018	0.91	0.30
Measles	1987	1993	6466.72	3233.36
Mumps	1990	2003	484.35	242.18
Rubella	1989	2000	2183.56	1091.78
Meningitis A (10 µg + 5 µg)	2010	2010	352.36	352.36
BCG	2001	2003	1822.24	1822.24
Influenza (Seasonal + Pandemic H1N1)			3.47	3.47

^a^ Data were collected from internal records of SII (Prasad S Kulkarni, personal communication). Well-organized data on the distribution of vaccine doses were available from 1996 onwards in Systems Application Products (SAP) data processing system. Data were assessed for each vaccine product. Figures between 1968 and 1996 were derived from Chartered Accountants (CA) certificates, which were part of the regulatory submissions to the Government of India (Prasad S Kulkarni, personal communication). This certificate gave data on the number of manufactured doses of the vaccines. Not all manufactured doses were distributed because of varying market demands. As a result, CA certificate figures were always higher than SAP figures since some doses were destroyed.

To estimate numbers of distributed vaccine doses, discount factors were applied to the manufactured doses in the period 1968–1996: 1.7% for DT vaccine, 2.06% for DTP vaccine, 2.93% for measles vaccine, 16.23% for MMR vaccine, 2.41% for rubella vaccine, 5.96% for Td vaccine and 0.54% for TT vaccine. These discount factors were based on data differences in the CA certificate figures and the SAP figures for each vaccine for the 1996–2018 figures. (Prasad S Kulkarni, personal communication).

^b^ Numbers of doses of each vaccine were segregated for each vaccine antigen. For example, for diphtheria antigen, doses of DT, Td, DTP, DTP-HepB, DTP-Hib, and DTP-HepB-Hib were cumulated. Numbers of fully immunized individuals were calculated by dividing the number of distributed doses with the required number of doses for protection for each antigen. As per WHO immunization schedule, five doses were required for rabies vaccine; three doses for DTP, polio, rotavirus, HepB, and Hib vaccines; two doses for MMR vaccines; and one dose for BCG, meningococcal A conjugate, and influenza vaccines.^37^

Abbreviation: HDC, human diploid cell

## Impact of SII vaccines on mortality

The UNICEF website provides information on the total number of doses procured by the agency during 1996–2016 (Table 2). A study by Ozawa et al. estimated that 10 vaccines supported by the Global Alliance for Vaccines and Immunization (GAVI) in 73 low- and middle-income countries will have prevented 500 million cases of illness, 70 million hospitalizations, 9 million cases of long-term disability and 20 million deaths during 2001–2020.^25^ Among the six vaccines presented in Table 3, SII has supplied all vaccines except the rotavirus vaccine so far. Yet only measles, rubella and meningitis A are considered here because SII is the biggest supplier for these vaccines. Assuming that 90% of the supplies were from SII, averted deaths as a result of SII vaccines reach almost 5.3 million in these 73 countries alone (Table 4).

**Table 2.** Total numbers of doses for each vaccine antigen procured by UNICEF for period 1996–2016^a38^

Antigen	Total doses procured (in millions)
BCG	2391
Diphtheria	3388.7
Tetanus	5979.7
Pertussis	3145.5
HepB	2398.7
Hib	1544
Measles	3918.5
Mumps	83.7
Rubella	667.6
Polio (tOPV + bOPV + mOPV + IPV)	33135
Meningitis	415.6

^a^Numbers of doses of each vaccine were segregated for each vaccine antigen. For example, for diphtheria antigen, doses of DT, Td, DTP, DTP-HepB, DTP-Hib, and DTP-HepB-Hib were cumulated.

**Table 3.** Estimated economic and health benefits of vaccinations in 73 GAVI-supported low- and middle-income countries, 2001–2020^a^^25^

Pathogen	Mortality and morbidity averted, 2001–2020^a)^				DALYs averted (millions)^b)^	Total averted costs of illness (billions of US$)^c)^
	Deaths (thousands)	Cases (millions)	Long-term disability (thousands)^c)^	Acute disease hospitalizations (millions)		
HepB	7 200	120	N/A	0.5	210	49.5
Hib	2 700	83	4 900	25	180	63.7
Measles	5 100	210	350	21	310	142
NmA	470	3.1	380	1.4	23	8.6
Rotavirus	390	21	N/A	0.7	25	8.8
Rubella	280	0.9	270	0.5	26	5.1
Total	20 000	500	9 200	70	960	347.1

^a^Estimates rounded.

^b^Expressed in 2010 values.

^c^Includes treatment costs, transport costs, lost caregiver wages, productivity loss due to disability, and productivity loss due to death.

**Table 4.** Estimates of SII vaccines impact on various parameters between 2001 and 2020

Pathogen	Mortality and morbidity averted				DALYs averted (millions)	Averted costs of illness
	Deaths (thousands)	Cases (millions)	Long-term disability (thousands)	Acute disease hospitalizations (millions)		Total (billions of US$)
Measles	5 100	210	350	21	310	142
NmA	470	3.1	380	1.4	23	8.6
Rubella	280	0.9	270	0.5	26	5.1
**Total**	**5.85 million**	**214**	**1 million**	**22.9 million**	**359 million**	**155.7 billions of US$**
**Estimated SII total**	**5.27 million**	**192.6 million**	**0.9 million**	**20.6 million**	**323 million**	**140.13 billions of US$**

### Measles

During 2000 and 2016, the number of estimated measles deaths declined by 84%, from 550,100 in 2000 to 89,780 in 2016 (Table 1). Compared with no measles vaccination, measles vaccination prevented an estimated 20.4 million deaths during 2000–2016^26^ and the total number of measles deaths prevented during 1990–2016 is near to 25 million [F. Marc LaForce, Personal communication]. The SII measles vaccine has been supplied to UNICEF and PAHO since 1992. Since the late 1990s, SII is the largest supplier of measles vaccine globally with 90% share of total supplies. If 90% of the deaths prevented during 1990–2016 are considered as the result of SII vaccines, the number of averted deaths globally reaches almost 22 million.

### Pertussis

In 1999 an estimate of 30 million pertussis cases and 390,000 deaths worldwide occurred in children under 5 years.^27^ Applying a similar model, an estimate of 24 million cases and 160,000 deaths from pertussis in children under 5 years occurred in 2014.^28^ It is therefore conceivable that in the 15 years between 1999 and 2014 hundreds of thousands of deaths in infants have been averted by improved vaccine coverage.

### Tetanus

A study by Kyu et al. calculated a total of 56,743 deaths due to tetanus in 2015 with 19,937 deaths in neonates and 36,806 deaths in older children and adults.^29^ In 1990 337,022 deaths were calculated with 199,118 in neonates and 137,904 in non-neonates. Between 1990 and 2015, the global mortality rate due to neonatal and non-neonatal tetanus declined by 90% and 81%, respectively.^29^

### Diphtheria

According to WHO, 97,164 diphtheria cases were estimated in 1980 declining to 8,819 in 2017.^30^ To a large part this remarkable decline of more than 90% was due to improved immunization programs. In last two decades, SII has also been the largest supplier of DTP vaccine to UNICEF and PAHO. Therefore, marked improvements in the global burden of diphtheria, tetanus, and pertussis are largely because of SII vaccines.

### Meningitis A

Meningitis A was a major health issue in the meningitis belt in Sub-Saharan Africa. Although meningococcal polysaccharide vaccines had been used during meningitis outbreaks, their impact on the population was negligible because they were deployed towards the end of the outbreaks.^31^ The licensing of the MenAfriVac by SII in 2010 lead to the launch of mass immunization campaigns in the meningitis belt. It has been estimated that this will have saved 470,000 lives and prevented 3.1 million cases of acute meningitis and 380,000 cases of long-term disability between 2011–2020.^25^

### Rubella

The financial burden of congenital rubella syndrome cases (CRS) has been estimated between $4,200 and $57,000 per case annually in middle-income countries and up to $140,000 over a lifetime in high-income countries.^32^ Globally, the estimated number of CRS cases decreased from 119,000 cases in 1996 to 105,000 cases by 2010. In the Americas, where rubella immunization is widespread, CRS cases decreased from 11,000 to 2,500 cases between 1996 and 2000, and <1 case in 2010.^33^ As a corollary, elimination of measles. rubella and CRS was accomplished in the whole of Latin America by 2017. SII was acknowledged as critical contributor to this success.^19^

## Summary

Over the last 50 years, SII has distributed billions of doses of life-saving vaccines, almost all of them to low-income countries through UNICEF, PAHO or other agencies. This has become possible because of the affordable cost and huge scale operations of SII vaccines.

SII supplied all important vaccines in the EPI programmes of the developing world. In the absence of precise data about epidemiology, mortality rates, vaccination coverage, etc., it is hard to determine the exact mortality and morbidity rates averted by SII vaccines. Yet, it is beyond doubt that millions of deaths have been prevented by SII vaccines.

## Limitations of data review

There are some limitations to these estimates which have been dealt with as follows: First, numbers of distributed vaccines before 1996 are not fully exact because of lack of computerized registration. Accordingly, available figures were interpreted cautiously to avoid any overestimate. Second, contribution of SII to total numbers of vaccine doses is not precisely known. UNICEF procurement data combined with SII distribution figures strongly indicate that in the 20-year period from 1996 to 2016, SII supplied the largest proportion of doses to UNICEF. In contrast, PAHO data are not available in the public domain. Third, precise figures for deaths and cases as well as disability-adjusted life years (DALYs), long-term disabilities, hospitalizations, and cost of illness averted by vaccines during the period of SII supplies are not accessible uniformly for all vaccine types. Moreover, global figures are available only for measles (since year 2000). For other vaccines, data are accessible from 73 GAVI eligible countries since 2001. As a result, declines in incidences were reported here which do not provide exact information on deaths and cases averted.

Nevertheless, the data confirm that SII has become the largest supplier of vaccines globally in the last 50 years paralleled by profound reductions in cases and deaths caused by various vaccine-preventable diseases (Table 4). Accordingly, this endeavor has saved millions of lives and prevented millions of permanent disabilities and hospitalizations. In addition to markedly reducing the humanitarian burden, this has saved billions of dollars of treatment costs across the world.

## Future perspectives

The impressive success of immunization programs in reducing the burden of diseases is well recognized, but the potential of vaccines has not been fully appreciated: Vaccines not only protect the vaccinees – high vaccination coverage but also protects against spread to the rest of the population by generating herd immunity. Moreover, resistance against vaccines has never developed under immune pressure induced by immunization. On the contrary, studies with pneumococcal vaccines have revealed that vaccination programs both in the North (US) and the South (South Africa) have led to reduced pneumococcal drug resistance.^34,35^

Even though vaccines are considered one of the most effective measures in medicine, R&D, production, and distribution remain costly. Nevertheless, increased investment into R&D for vaccines against diseases that cannot be tackled by immunization yet is urgently needed. At the same time, an affordable price for current vaccines is equally important for further reduction of the threat of infectious diseases. The example of SII demonstrates that vaccine production at affordable cost is feasible if large quantities of doses are being purchased.^3-6^ There are other vaccine manufacturers from developing and emerging countries which have started to supply affordable vaccines in recent past, though they do not have the broad portfolio of prequalified vaccines supplied by SII. A list of members of the developing country vaccine manufacturers network (DCVMN) can be found on their website.^36^ This network includes both private and public-owned companies. The SII model can be replicated and scaled up but this will require multiple factors including visionary leadership, philanthropic management philosophy, highly skilled and experienced scientists, willingness to invest financial and other resources in high-risk vaccine development projects, as well as strict compliance to the regulatory and quality standards. Since this model has worked very well over the last 50 years, SII can most likely sustain this model, notably because of the increased awareness of the role of vaccines in improved global health and because of the need for new and improved vaccines for global health programmes. Therefore, SII can serve as role model for new initiatives to take care of regional production and supply of affordable vaccines.

Public immunization programs are expensive since huge amounts of doses must be provided to healthy individuals to prevent disease in only a proportion of these recipients. Yet, vaccines represent an exuberant benefit to society. It is for this reason that nongovernmental and intergovernmental organizations such as GAVI, UNICEF, and PAHO have been created. Public-private-philanthropic partnerships have proven a valid model of continued production of highly affordable vaccines for universal immunization of every child, both in the North or the South.
